# Obituary - John Payne (Jack) Woodall, Co-founder of ProMED-mail

**DOI:** 10.11604/pamj.2016.25.123.11053

**Published:** 2016-10-29

**Authors:** Raoul Kamadjeu

**Affiliations:** 1 Managing Editor, Pan African Medical Journal, Nairobi, Kenya

**Keywords:** John Jack Woodall, Promed-Mail, Yellow fever, infectious diseases

## Obituary

**Figure:**
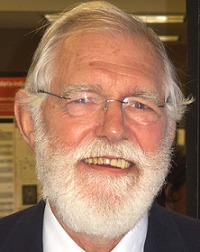


It is with great sadness that I heard of the demise of John (Jack) Woodall co-founder of ProMed-Mail [1]; it happened on the evening of Monday 24 Oct 2016 in London.

The news struck through a ProMed-Mail alert; just like one of the many I received from John during the yellow fever outbreak in Angola. He always forwarded me the latest post on yellow fever accompanied with the mention “Raul, look at this – this outbreak will be big”. Despites my many insistence, he just couldn’t write “Raoul”; a sign maybe of the long time he spent in Latin America.

I remember when he shared the article he co-authored on the effectiveness of fractional doses of yellow fever vaccine [2]; it came with the message “Raul, make sure your boss reads this – this is a game changer!!”. When the SAGE recommended the use of fractional doses, he was delighted and told me “They did it, now they should move quickly”. I felt honored that John could find time to entertain my questions; we discussed at length about his experience fighting yellow fever in Africa and Latin America. I am reading once again and with deep pain the editorial I convinced him to write for PAMJ in May this year [3].

Jack worked for CDC, WHO and various other organizations; he conducted intensive research on yellow fever and other exotic viruses in many countries in Africa and Latin America. The detailed and entertaining carrier path of John, in his own words, can be found here [4].

I will miss this great man, his sense of humor and the precious advices he provided.
